# The effect of constructing versus solving virtual patient cases on transfer of learning: a randomized trial

**DOI:** 10.1007/s40037-015-0242-4

**Published:** 2016-01-11

**Authors:** Martin G. Tolsgaard, Rikke M. H. G. Jepsen, Maria B. Rasmussen, Lars Kayser, Uno Fors, Lars C. Laursen, Jesper H. Svendsen, Charlotte Ringsted

**Affiliations:** 1 Centre for Clinical Education, Capital Region of Denmark and University of Copenhagen, Blegdamsvej 9, 2100 Copenhagen, Denmark; 2 Department of Obstetrics and Gynaecology, Nordsjælland University Hospital, Hillerød, Denmark; 3 Danish Institute for Medical Simulation, Herlev University Hospital, Capital Region of Denmark and University of Copenhagen, Copenhagen, Denmark; 4 Faculty of Health Sciences, University of Copenhagen, Copenhagen, Denmark; 5 Department of Computer and Systems Sciences, Stockholm University, Stockholm, Sweden; 6 Department of Pulmonary Medicine, Herlev University Hospital, Herlev, Denmark; 7 Department of Cardiology, Department of Clinical Medicine, The Heart Centre, Rigshospitalet, Copenhagen University Hospital, University of Copenhagen and Danish Arrhythmia Research Centre, University of Copenhagen, Copenhagen, Denmark; 8 Faculty of Health, Aarhus University, Aarhus, Denmark

**Keywords:** Virtual patients, Simulation-based medical education, Learning, Transfer of learning

## Abstract

The purpose of this study was to explore the effect of actively constructing virtual patient (VP) cases compared with solving VP cases on knowledge gains, skills transfer and time spent on cases. Forty-five fourth-year medical students were randomized to constructing (VP-construction, *n* = 23) or solving (VP-solving, *n* = 22) four cardiopulmonary VP cases. Whereas the VP-solving group solved the cases, the VP-construction group only received the final diagnosis and had to complete the history, physical findings, and lab results. After a week, participants completed a transfer test involving two standardized patients representing cardiopulmonary cases. Performances on the transfer test were video-recorded and assessed by two blinded raters using the Reporter, Interpreter, Manager, Educator (RIME) framework. Thirty-nine participants completed the transfer test. The VP-construction group spent significantly more time on the VP cases compared with the VP-solving group, *p* = 0.002. There were no significant differences in RIME scores between the VP-construction group and VP-solving group, *p* = 0.54.

In conclusion, engaging novice students in active VP case construction may be more time consuming than solving VP cases, without resulting in superior skills transfer.

## Essentials

- Engaging novice students in active VP case construction is more time consuming than solving VP cases.
- The extra time consumed does not result in superior knowledge gains or improved skills transfer for students who were instructed in constructing their own VP cases.
- Future research may focus on whether more advanced learners may benefit from increasing levels of self-guided learning during practising with VPs as well as the role of self-guided learning as preparation for future learning.

## Introduction

Over the past decades, virtual patients (VPs) have been increasingly used to expose learners to multiple and varied patient cases. Virtual patients can be defined as *‘an interactive computer simulation of real-life clinical scenarios for the purpose of health care and medical training, education or assessment’* [1]. Their educational effect is presumably exerted through stimulating knowledge acquisition and application in order to arrive at a diagnosis or a management plan [2]. VPs are associated with large positive effects on knowledge, clinical reasoning, and skills when compared with no training and non-inferior effectiveness when compared with non-computer instruction [3, 4].

Although the effectiveness of VPs has been established, questions still remain regarding when and how best to use then. VPs provide structured representations of patient cases that may be useful as an adjunct to the unstructured and opportunistic learning in the clinical setting [5]. The highly structured representation of patient cases does not, however, reflect the management of real patients causing learners to perceive the interaction as too constructed [6, 7]. On the other hand, there is evidence that providing less structured environments and activities may engage learners in more effective cognitive processes for learning. Traditional VP learning engages learners in activating existing knowledge, assimilating or storing new information and is therefore considered a form of active learning. However, it does not necessarily prompt learners to construct activities that go beyond the presented material [8]. In order to engage learners in *constructive* activities, [8] VPs should prompt learners to produce mental models of the information to be learned by linking new knowledge with existing knowledge in order to elaborate, justify and provide reasons for their choices. However, the highly structured step-by-step design of traditional VP cases may not support these types of activities as, for example, when learners instead skip or disengage with the case [7]. Designing VP cases as constructive activities, where learners build mental models based on less structured platforms, may therefore produce superior learning outcomes than highly structured step-by-step VP cases.

Excessive instruction during performance can be detrimental to learning [9]. In fact, under some conditions self-guided and self-regulated learning may provide superior learning outcomes when compared with instructor-regulated learning [10–12]. Engaging learners in self-regulated practice may stimulate metacognitive activities that enable learners to identify their own learning needs and monitor their performance. These strategies for active learning have previously been associated with improved transfer [13]. Hence, providing opportunities for self-regulated learning under adequate external guidance may improve the learning outcomes of practising with VPs. Engaging learners in self-regulated practice using constructive activities may stimulate deeper cognitive processing that may result in improved knowledge gains as well as transfer of learning [13]. The aim of this study was therefore to explore the effect of actively constructing VP cases compared with solving VP cases on knowledge gains and skills transfer in a group of pre-clerkship medical students.

## Methods

### Study design

**Fig. 1:**
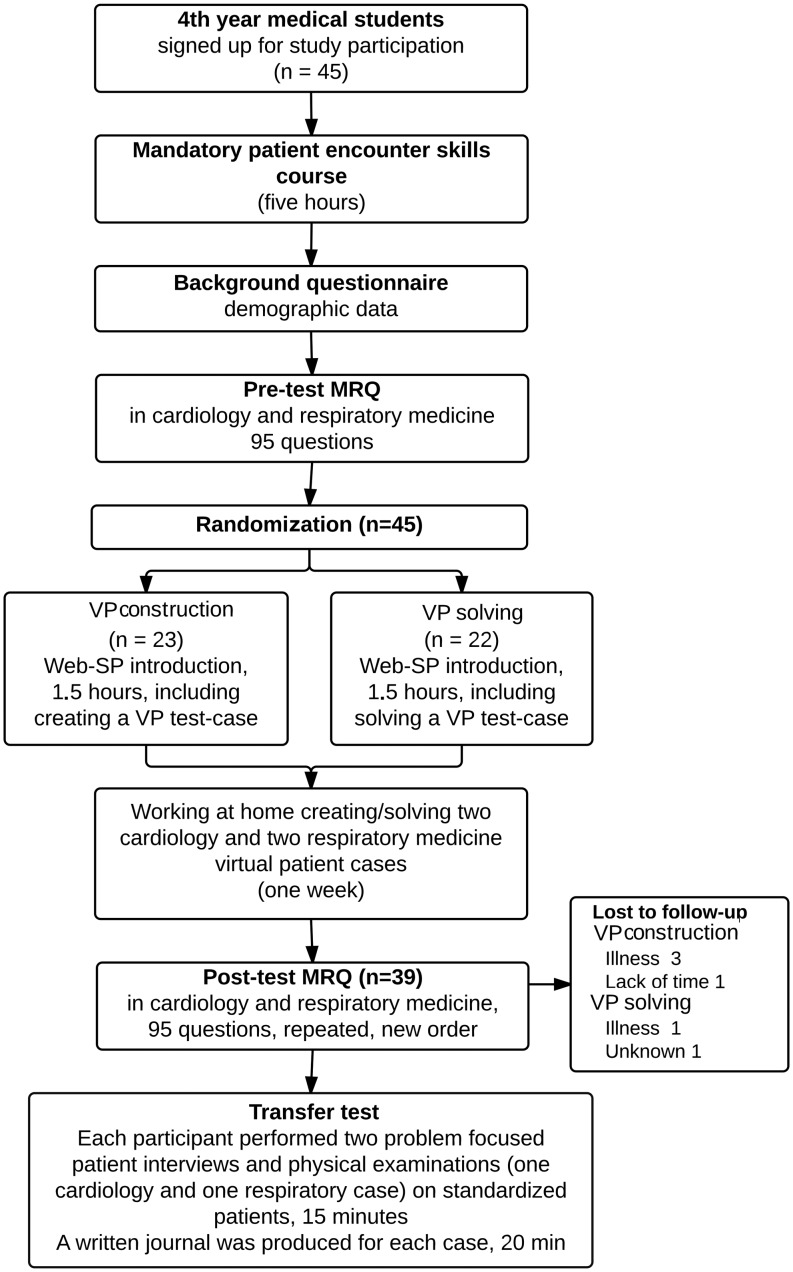
Flowchart of the study. Participants were randomized to VP-construction or VP-solving

This study was a controlled observer-blinded superiority trial conducted between 29 January 2012 and 8 May 2012 and reported according to the CONSORT statement. Ethical approval was granted in terms of an exemption letter (Protocol No. H-1-2013-FSP-6) and the study was registered with clinicaltrials.gov (Clinical Trials Identifier NCT02400606). The study flowchart is illustrated in Fig. 1.

### Setting and participants

The study was carried out in the Centre for Clinical Education, Copenhagen University Hospital Rigshospitalet, Capital Region, Denmark. The medical programme at our university consists of a six-year traditional curriculum with basic science teaching during the first three pre-clerkship years and clinical sciences during the last three clerkship years. Before entering clinical clerkships, all students must complete a mandatory five-hour course in patient encounter skills.

Participants included pre-clerkship fourth-year medical students from the University of Copenhagen. Students were recruited through an advertisement in the student newsletter and eligible participants included those who had completed the mandatory course on patient management skills and were about to enter their first clinical clerkship. Participants were informed that they would get access to a virtual patient training platform and subsequently be allowed to practise their skills with standardized patients in the simulated environment. All participants provided informed consent to participate and received financial compensation for their participation (60 USD). Participants were enrolled on a first come first serve basis until the required sample size was reached. Participants were randomized using a computer-generated list of random numbers to constructing VPs (VP-construction) or solving VPs (VP-solving) in a 1:1 ratio.

### The VP system and cases

The VP Programme used for this study was Web-SP®, version 3.3 Stockholm University, Stockholm, Sweden [14, 15]. The VP programme is an explorative linear-interactive system that is accessed online. The Web-SP programme has been described in further detail elsewhere; [15] in brief it involves gathering information from patient interviews and physical exam findings in order to arrive at a diagnosis and management plan. The English version of the programme was translated into Danish by one of the study authors [RMJ]. The VP cases were based on four real patients and the results from their laboratory and clinical tests. The diagnoses of the four cases included (1) exacerbation of chronic obstructive pulmonary disease (COPD), (2) acute severe asthma, (3) atrial fibrillation, and (4) ST-elevation myocardial infarction (STEMI). The final cases were assessed and proofread by two consultants in cardiology and respiratory medicine. A pilot study was performed to assess feasibility of the study design and the instructional material provided to the participants as well as to provide data for the sample size calculations. The pilot study included four medical students in each group, who underwent a pre- and post-test multiple-response questionnaire (MRQ) and a transfer test involving two standardized patients. Instructional material was revised based on the responses and comments of participants in the pilot study.

### Interventions

All participants completed one practice case to become familiar with the VP system before completing four VP cases. For each of these four cases all participants were presented with a case overview including an admission note from the patient’s family physician as well as brief notes recorded by the emergency unit nurse including patient vital signs. The VP-construction participants were informed about the patient’s final diagnosis. From this diagnosis, the VP-construction participants were instructed to construct the patient case using the *‘teacher-mode’* settings in the Web-SP programme allowing participants to edit the case. VP-construction participants were carefully instructed only to add information relevant to the case and not to spend time on writing task-irrelevant information. According to what could be expected for each VP case, VP-construction participants filled out relevant history, physical findings, laboratory, and diagnostic imaging results. VP cases in the VP-construction group were preset to show normal findings and participants had to actively change relevant clinical and paraclinical findings.

VP-solving participants were instructed to solve each of the four VP cases by clicking through the relevant information. They were not informed of the final diagnosis but were instructed to provide a tentative diagnosis, differential diagnoses, and a management plan as well as to justify their choices. None of the participants received any feedback on their performance regarding constructing or solving VP cases. All participants were allowed to use whatever learning material they wished but were required to register the type of materials used and amount of time they spent studying. The participants were instructed on how to use the system during a briefing on day 1 and completed solving or constructing VP cases at home during the next week. All participants were blind to the study hypothesis.

### Outcomes

The main outcome measure was transfer test performance a week after the VP system had been introduced to the study participants. The transfer test included managing two encounters with standardized patients in a simulated setting. The participants had 15 min available to take each standardized patient’s history and to do a physical examination. They were provided with abnormal findings from the physical examination as well as ECG and lab results and were instructed to fill out the patient records after each encounter. The two standardized patients were instructed to portray a case of community-acquired pneumonia and stable angina pectoris, respectively. All performances were video-recorded to allow subsequent assessment by two blinded physicians. Performances were rated using a scoring form related to the Reporter, Interpreter, Manager, Educator (RIME) framework. The RIME framework is widely used for in-training evaluation of medical students’ patient encounter skills. The RIME framework is often used to describe the development of skills from being a ‘reporter’ (obtains history and performs physical examination), ‘interpreter’ (interprets findings correctly), ‘manager’ (manages the patient based on interpretation of findings), to ‘educator’ (teaching one’s self and others). Validity evidence of the RIME framework regarding content, response process, relations to other variables, and internal structure has been explored in previous studies [16, 17].

Secondary outcome measures included knowledge gains measured through pre- and post-test multiple-response questionnaires (MRQs). Validity evidence regarding MRQ content and internal structure was achieved by pilot testing its 95 questions in a group of eight pre-clerkship medical students and two consultants in respiratory medicine and cardiology. Participants’ satisfaction with the VP experience and time used during VP practice were assessed in a post-training questionnaire using 5-point and 9-point Likert scales, respectively.

### Sample size calculations

The pilot test RIME scores were used to determine the sample size for the study. Mean RIME scores for pilot group participants who constructed and solved cases were 35.9 % (SD 7.7) and 28.4 % (SD 5.8), respectively. Using an alpha level of 0.05, 38 participants were needed to achieve a power of 90 %.

### Statistics

MRQ and RIME scores were calculated into percentages of maximum scores. RIME scores were calculated as the mean scores of the two raters. Kurtosis and skewness were determined for all data to assess normal distribution and means were calculated for all scores. RIME scores were compared using Student’s t-test and MRQ scores were calculated using a factorial 2 × 2 repeated-measures ANOVA. Internal consistency of MRQ and RIME scores was assessed using Cronbach’s alpha and for the RIME scores inter-rater reliability was determined using intraclass-correlation coefficients (ICC). Chi-square tests were used to compare participants’ use of learning materials during VP practice.

## Results

Thirty-nine participants completed the transfer test as well as the pre- and post-test MRQs (Fig. 1). Baseline characteristics of study participants are shown in Table 1. There were no significant differences in RIME scores between the VP-construction group (mean 60.8 %, SD 11.5) and VP-solving group (mean 59.1 %, SD 12.8), *p* = 0.54. Inter-rater reliability of RIME scores was an ICC of 0.57.

**Table 1:** Baseline characteristics of study group participants randomized to constructing (VP-construction) or solving (VP-solving) VP cases. There were no differences in any of the baseline characteristics between the two groups

Group	VP-solving (*n* = 20)	VP-construction (*n* = 19)
Age, median (range) years	24 (22–32)	23 (21–29)
Gender		
Male	50 % (10/20)	37 % (7/19)
Female	50 % (10/20)	63 % (12/19)
How much time do you use computers/tablets/smartphones (not for text messages or talking)/per day?		
< 1 h	15 % (3/20)	32 % (6/19)
1–2 h	50 % (10/20)	37 % (7/19)
2–5 h	25 % (5/20)	26 % (5/19)
> 5 h	10 % (2/20)	5 % (1/19)
Have you previously used computer-based education?		
Yes	55 % (11/20)	42 % (8/19)
No	45 % (9/20)	58 % (11/19)
Time from pre-test MRQ to post-test MRQ and transfer test, mean (SD) days	7.3 (1.7)	7.0 (1.4)
Use of learning materials during VP practice		
Textbooks	79 % (15/19)	95 % (18/19)
Online practice guidelines	16 % (3/19)	16 % (3/19)
Google, Wikipedia or Youtube	58 % (11/19)	32 % (6/19)
Government-supported webpages	42 % (8/19)	37 % (7/19)
Lecture or study notes	11 % (2/19)	11 % (2/19)

There were significant improvements in MRQ scores from pre- to post-test (mean 58.7 % SD 6.5 versus mean 62.0 % SD 5.4, *p* < 0.0001, partial eta squared = 0.40) but no interaction between scores and group (*p* = 0.19) or effect of group allocation (*p* = 0.84). Internal consistency of pre- and post-test MRQ was Cronbach’s alpha = 0.66 and 0.55, respectively, and test/re-test reliability was Cronbach’s alpha = 0.86. Performance scores on each RIME element and MRQ scores are shown in Table 2.

**Table 2:** Transfer test RIME scores and pre/post-test MRQ scores of students randomized to constructing (VP-construction) or solving (VP-solving) VP cases

Group	VP-solving (*n* = 20)	VP-construction (*n* = 19)	Effect size (Cohen’s D, 95 % CI)
Reporter	65.8 % (SD 12.0)	65.9 % (SD 10.8)	0.01 (− 0.62 to 0.64)
Interpreter	66.4 % (SD 17.4)	68.4 % (SD 16.7)	0.12 (− 0.51 to 0.74)
Manager	60.4 % (SD 15.2)	62.5 % (SD 13.4)	0.15 (− 0.49 to 0.77)
Educator	43.3 % (SD 22.0)	45.5 % (SD 15.7)	0.11 (− 0.52 to 0.74)
RIME score (total)	59.1 % (SD 12.8)	60.8 % (SD 11.5)	0.14 (− 0.70 to 0.55)
MRQ pre-test score	58.9 % (SD 7.0)	58.4 (SD 6.1)	0.08 (− 0.70 to 0.55)
MRQ post-test score	61.4 % (SD 5.2)	62.6 % (SD 5.7)	− 0.22 (− 0.41 to 0.85)

**Fig. 2:**
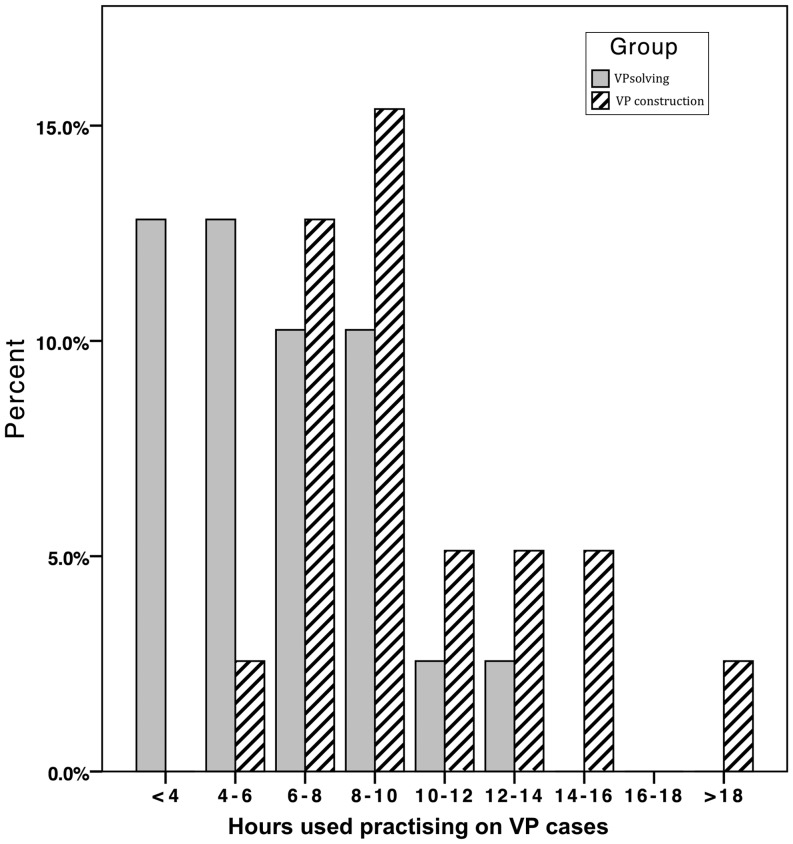
Time spent working with the VPs in the two groups of students, who constructed (VP-construction) or solved (VP-solving) four VP cases

The VP-construction group spent significantly more time (median 9 h, range 5–19) on the VP cases compared with the VP-solving group (median 6 h, range 2–13), *p* = 0.002 (Fig. 2). There were no differences in participant satisfaction with the VP system between VP-construction group (mean 4.0 SD 0.7) and VP-solving group (mean 4.1 SD 0.6), *p* = 0.46. There were no significant differences in participants’ use of learning recourses (textbooks, online resources, lecture notes) during their practice with the VP cases (*p*-values > 0.05, Table 1).

## Discussion

In this study, there was no effect of constructing VP cases as opposed to solving them on participants’ knowledge gains or transfer of skills. However, VP-construction participants spent about 50 % more time on the VP cases than the VP-solving participants.

Explanations for these results may be that practising with VPs failed to produce a differential effect on students’ learning or that our assessment instruments failed to capture performance differences between groups. Although there may be no significant differential effects of the two types of VP practice, there are indications that the interventions produced learning effects. First, significant improvements in MRQ scores after practising with VPs were demonstrated in the present study, consistent with existing evidence on the effectiveness of VP practice [2–4]. Second, the participants in the present study achieved markedly higher performance scores in the patient encounter skills test than year-four students in a previous study conducted under similar conditions except that no preparatory practice was allowed before managing standardized patients (mean 59.9 % versus mean 41.1 %) [17]. Finally, validity evidence from multiple sources supports the use of the RIME framework for assessing students’ patient management skills [16–18] and the required sample size was reached. The finding that there were no differences in knowledge gains or transfer test performances is therefore most likely to reflect equivalent learning gains rather than no learning or failure to detect differences between groups.

Another explanation for the observed results is that novice learners may need more structure and guidance as opposed to advanced learners. Research has shown that novices benefit from worked examples but that advanced learners need progressively more challenging problems and less structure [2, 19]. The finding that instructional techniques that work well with one group of learners can lose their effectiveness with learners at different levels of expertise is also termed the *‘expertise reversal effect’* [19]. In the context of our study, we may hypothesize that providing less structure and guidance for the VP-construction group was premature with respect to their learning levels, despite their prior training in patient encounter skills and introduction to working with VPs. The large spread in use of time on the VP cases in both groups may reflect heterogeneity in the ways participants interacted with the VP system as well as their learning needs. Hence, we cannot rule out that progressive levels of independence and decreasing amounts of external guidance may be an advantage for more experienced learners than those included in the present study.

Strengths of this study include the use of a randomized design, an assessment instrument with established validity evidence, assessor blinding, and use of a transfer test. There are also some limitations. Most importantly, we only evaluated participants’ knowledge gains and transfer test performances and we did not directly measure their clinical reasoning skills. Some researchers claim that the main effect of practising with VPs is mediated through improvement in reasoning skills, [2] which was only assessed through a transfer test in the present study. Relying on a transfer test may have caused some dilution of learning effects, [20] which ultimately increases the risk of type II errors. Moreover, the limited length of the intervention may have been insufficient to demonstrate small differences in learning outcomes between groups. However, potential differences below the magnitude that could be detected on a transfer test may not justify the large increases in use of time associated with constructing VP cases rather than solving them. The transfer test used in the present study can be considered a *transfer-in* test, in which participants applied their skills from the common learning resource (i.e. the VP cases they practised). We did not assess *transfer-out* in terms of participants’ ability to apply the knowledge and skills they attained from the common learning resource to a target transfer problem involving new cases and a new setting [21]. In other words, we did not assess whether constructing cases as opposed to solving them prepared participants better for future learning. In an attempt to clarify the ‘blackbox’ of successful transfer of learning, this study contributes by identifying what does and does not work when practising with VPs. Future qualitative studies may help clarify which factors are perceived as useful or detrimental to learning during VP case construction as well as application of knowledge and skills from VP cases to patient encounter management.

In conclusion, engaging novice students in active VP case construction is more time consuming than solving VP cases but does not result in superior knowledge gains or improved skills transfer. Future research may focus on whether more advanced learners may benefit from increasing levels of self-guided learning during practising with VPs as well as the role of self-guided learning as preparation for future learning.
